# The Personal Element

**Published:** 1891-08-15

**Authors:** 


### Passing Topics.

### THE PERSONAL ELEMENT.

It is only natural that a consideration of the several subjects treated of in the remarkable evidence given before the Lords' Committee by Mr. Burdett should develop differences of opinion. Yet everyone interested in hospital work must feel a satisfaction that the inquiry did not close without an opportunity being given so redoubtable a witness to set forth some, at least, of the conclusions arrived at by one whose acquaintance with the details of hospital administration has been acquired not alone by utilising the ordinary channels of experience, but by such far-reaching and self-imposed labours and investigations as may well be accounted unique.

If, in dealing with evidence covering so wide an area, and containing much that is either actually original or original in the manner of its presentment, the hospital experts, and those who think themselves of that order, will be at no loss to find material for discussion, we are disposed to believe that upon one point, at any rate, there will be on their part something approaching to unanimity. Mr. Burdett probably only gave expression to the conviction of ninety-nine out of every hundred people who have lent their minds to the subject when he pronounced the committee as a weak factor in hospital administration, and proceeded to point out the value of personal service.

When we assert that committees generally are woefully inefficient, we readily admit that there are some well deserving of exemption from the stricture. But we opine that these exceptions are only numerous enough to prove the rule and to show what a committee ought to be, and can be, if rightly constituted and composed of suitable members. It is the fashion, unfortunately, to assume that anybody of a certain social position is eligible to serve upon a hospital committee, and that the hospital should not only take his efficiency for granted, but should be grateful for such intermittent and inefficient service as he can render, because it is not paid for. Here we have an attempt to support an unreasonable contention by a subtle incoherence which obscures the real issue. That the argument answers the purpose may be admitted, inasmuch as a majority of people only too unquestionably acquiesce in the preposterous sentiment that a man who voluntarily accepts office without remuneration is under no obligation to perform the duties attaching to such office, for the reason that his services are not remunerated.

In the case of hospital committees, the due performance of duty manifestly requires an exercise of the personal interest and sympathy Mr. Burdett demands. When these qualities are forthcoming, and not until then, knowledge and aptitude follow, and the institution gets the benefit. We confess we are not very hopeful that the committee system will ever prove equal to all that is required of it, because the rendering of continuous service without a fixed individual responsibility, and without the impetus communicated by the force of novel and exciting circumstances, is with most people an ungrateful task. Concern naturally has a tendency to evaporate when it is distributed, and sympathy when shared by many is often too attenuated for recognition. But until a revolution occurs in things charitable for which we are not yet prepared, committees will be indispensable to our institutions and it is just as well therefore considering their importance that when weighed they should not be found wanting.

